# The impact of doxycycline on human contextual fear memory

**DOI:** 10.1007/s00213-024-06540-w

**Published:** 2024-02-09

**Authors:** Jelena M. Wehrli, Yanfang Xia, Aslan Abivardi, Birgit Kleim, Dominik R. Bach

**Affiliations:** 1 Department of Psychiatry, Psychotherapy, and Psychosomatics, Psychiatric Hospital, University of Zurich, Lenggstrasse 31, 8008 Zurich, Switzerland; 2 Wellcome Centre for Integrative Neuroimaging, Nuffield Department of Clinical Neurosciences, University of Oxford, FMRIB Building, John Radcliffe Hospital, Headington, Oxford, OX3 9DU UK; 3 Wellcome Centre for Human Neuroimaging, University College London, Russell Square House, 10-12 Russell Square, London, WC1B 5EH UK; 4 Hertz Chair for Artificial Intelligence and Neuroscience, Transdisciplinary Research Area Life & Health , University of Bonn, Am Probsthof 49, 53121 Bonn, Germany

**Keywords:** Fear memory, Memory consolidation, Memory modification, Doxycycline, MMP inhibition

## Abstract

**Rationale:**

Previous work identified an attenuating effect of the matrix metalloproteinase (MMP) inhibitor doxycycline on fear memory consolidation. This may present a new mechanistic approach for the prevention of trauma-related disorders. However, so far, this has only been unambiguously demonstrated in a cued delay fear conditioning paradigm, in which a simple geometric cue predicted a temporally overlapping aversive outcome. This form of learning is mainly amygdala dependent. Psychological trauma often involves the encoding of contextual cues, which putatively necessitates partly different neural circuits including the hippocampus. The role of MMP signalling in the underlying neural pathways in humans is unknown.

**Methods:**

Here, we investigated the effect of doxycycline on configural fear conditioning in a double-blind placebo-controlled randomised trial with 100 (50 females) healthy human participants.

**Results:**

Our results show that participants successfully learned and retained, after 1 week, the context-shock association in both groups. We find no group difference in fear memory retention in either of our pre-registered outcome measures, startle eye-blink responses and pupil dilation. Contrary to expectations, we identified elevated fear-potentiated startle in the doxycycline group early in the recall test, compared to the placebo group.

**Conclusion:**

Our results suggest that doxycycline does not substantially attenuate contextual fear memory. This might limit its potential for clinical application.

**Supplementary Information:**

The online version contains supplementary material available at 10.1007/s00213-024-06540-w.

## Introduction

Remembering adverse outcomes can help to avoid them in the future and thus serve to ensure well-being and survival. However, such memories can also cause debilitating limitations to quality of life, such as in posttraumatic stress disorder (PTSD) after exposure to life threatening trauma (American Psychiatric Association 2013). The predominant treatments for PTSD are psychotherapy with trauma-focused exposure (Watkins et al. 2018) and symptomatic pharmacotherapy (Krystal et al. 2017). Many patients, however, experience only modest improvements with psychotherapy (Bisson et al. 2013; Bradley et al. 2005) or medication (Davidson et al. 2006; Stein et al. 2006). A possible underlying reason is that current treatment options conceptually aim to suppress unwanted responses but might not interfere with the actual aversive memory (Bouton 2004). With this in mind, preclinical research has focused on prevention (or modification) of traumatic memory, typically employing Pavlovian fear conditioning as an experimental model (LeDoux 2000; Pape & Pare 2010).

In Pavlovian conditioning, a neutral cue (conditioned stimulus, CS) is coupled with an aversive stimulus (unconditioned stimulus, US). Over several repetitions, this leads to conditioned responses (CR) to the presentation of the CS alone. To retain the association between CS and US over time, synaptic reconfiguration leading to long-term potentiation (LTP) of amygdala neurons is necessary (LeDoux 2000). Thus, inhibition of fear memory consolidation could potentially be achieved by interfering with signalling pathways that are involved in inducing LTP. The extracellular enzyme matrix metalloproteinase 9 (MMP-9) is an essential molecule in these pathways (Beroun et al. 2019; Huntley 2012), and LTP can be reduced by inhibiting MMP-9 (Gorkiewicz et al. 2015; Nagy 2006; Wang et al. 2008). Blocking MMP-9 has also been shown to reduce learning in animals (Meighan et al. 2006; Wright et al. 2007). Although there are no specific MMP-9 inhibitors currently approved for use in humans, the antibiotic doxycycline inhibits MMP-9 amongst other MMPs (Golub et al. 1991; Hanemaaijer et al. 1998; Kim et al. 2005) and has already been shown to reduce memory retention, when applied before conditioning in a Pavlovian cued fear conditioning paradigm (Bach et al. 2018)

Taken together, these preclinical findings may motivate translation into a clinical intervention for prevention of PTSD after trauma. However, cued fear conditioning is a reduced model of realistic adverse events. Before use in clinical populations, it would be desirable to test the effect of the intervention in experiments that model other aspects of learning, such as memory for the context that surrounds a trauma, which is clinically important (Al Abed et al. 2020; Liberzon & Abelson 2016; Spence et al. 2019). While cue conditioning involves plasticity in the amygdala for successful learning (Davis & Whalen 2001; LeDoux 2000; Maren 2001), contextual learning is more complex and involves more extensive neural circuits. It has been suggested that depending on the experimental parameters, contextual learning can be supported by two types of representations, elemental and configural associations (Rudy 2009; Rudy et al. 2004). Elemental associations link independent representations of individual features of the context with an event, assumed to be supported by the neocortex (Rudy et al. 2004). Configural representations on the other hand bind multiple contextual features into a single representation of the context, which is thought to be hippocampus dependent (Maren et al. 2013; Rudy 2009).

In the present study, we sought to investigate the impact of doxycycline on contextual conditioning in a randomized placebo-controlled double-blind trial. We employed a configural conditioning task that seeks to maximize configural associations (Stout et al. 2018, 2019), thus suggesting hippocampal involvement. In a preceding methodological investigation, we demonstrated successful fear memory recall after 7 days in this paradigm (Xia et al. 2023).

## Materials and methods

### Participants

**Fig. 1:**
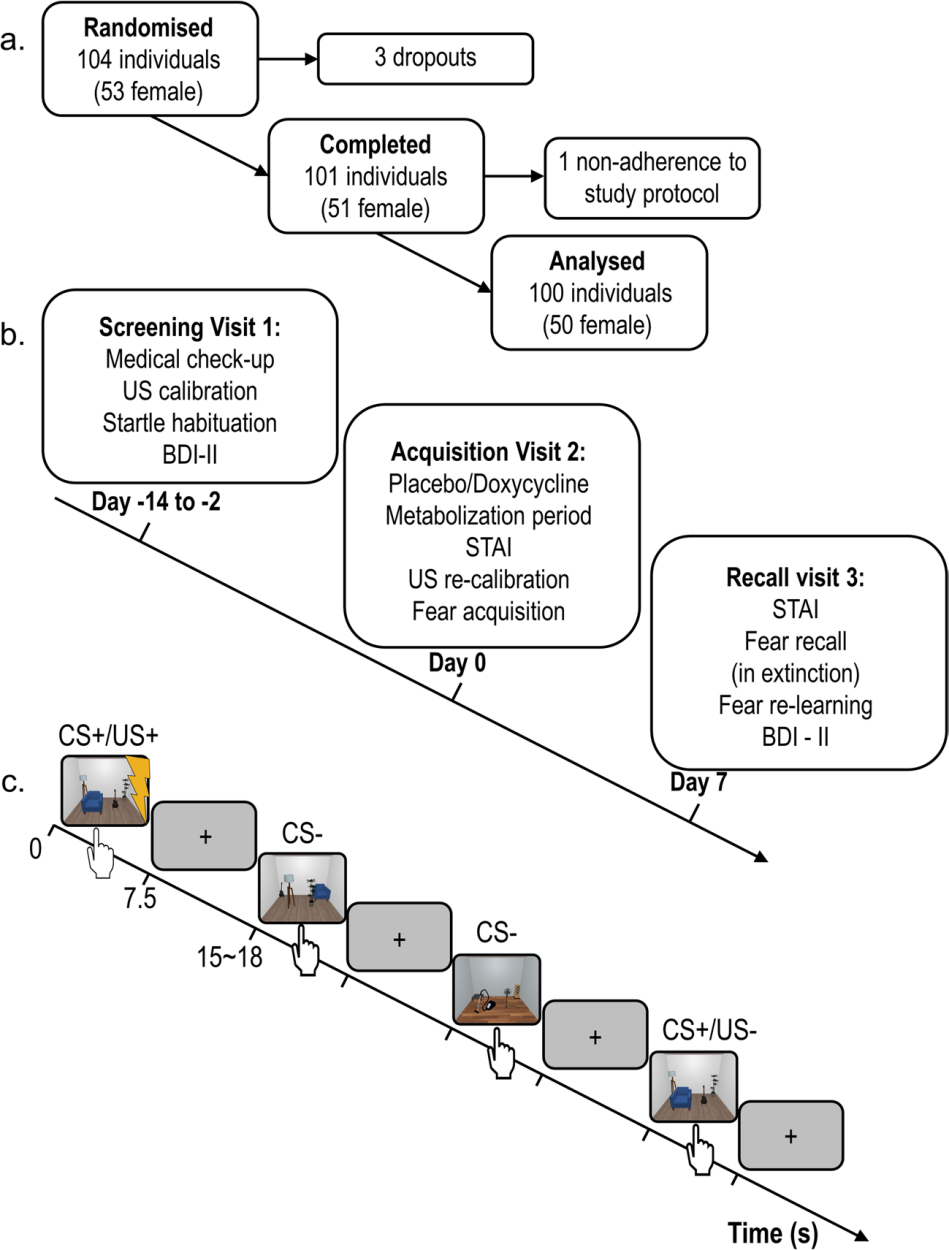
Experimental protocol. **a**: Recruitment and exclusion of participants, for details on sample characteristics see supplementary information Table S1. **b**: Study timeline. **c**: Intra-trial procedure: a static room picture was presented for 7.5 s, 83% of CS + co-terminated with a 500-ms electrical stimulation (US +), inter-trial interval was jittered between 7.5 and 10.5 s

One hundred and four participants were recruited from the general population between 18 Mar 2021 and 13 Dec 2021 and were randomly assigned to placebo (*n* = 53, 27 females) or doxycycline (*n* = 51, 26 females). Because of similarities in procedure, participants in a previously reported RCT with doxycycline (Wehrli et al. 2023) were excluded from participation in the present study. Two participants (both doxycycline) did not complete visit 2 per protocol due to vomiting shortly after ingestion of the drug. One further participant (placebo) did not attend visit 3 as they were obliged to self-isolate during the COVID-19 pandemic. One participant (doxycycline) was excluded from all analyses, because they later revealed prior participation in the preceding methodological experiment with the same paradigm and CS. The final reported sample therefore comprises 100 participants; *n* = 52 in the placebo group (26 females) and *n* = 48 in the doxycycline group (24 females) (see Fig. 1a and for details supplementary information (SI) Table S1). Three participants were excluded from analysis of the re-learning phase only, due to equipment malfunction.

The study was approved by the governmental research ethics committee (Kantonale Ethikkomission Zürich KEK-ZH-2018–01973) and the Swiss Agency for Therapeutic Products (Swissmedic, Bern, Switzerland; 2019DR1026) and was conducted in accordance with the Declaration of Helsinki. Before the experiment, all participants gave written informed consent with a form approved by the ethics committee. The study was pre-registered with a WHO-approved primary registry (German Clinical Trials Register, DRKS00017037) and at the Swiss Federal Complementary Database (Kofam: SNCTP000003485).

### Power analysis

We conducted a power analysis in G*power to determine required sample size. In a preceding methodological study, the effect size to distinguish CS + /CS- in cued fear conditioning using startle eye-blink responses (SEBR) in a control group was (Cohen’s) *d* = 1.17 (Khemka et al. 2017). Assuming equal variance under doxycycline, a 50% reduction in fear memory would correspond to an effect size of *d* = 0.59 (Bach et al. 2020). To achieve 80% power with an alpha rate of 0.05 required a sample size of *N* = 74. To account for unknown variability of the intervention, we planned to recruit *N* = 100.

After the trial protocol was registered and recruitment for the present study commenced, we conducted two methodological experiments to investigate the effect size for memory retention in the employed configural fear conditioning protocol (Xia et al. 2023). These experiments revealed effect sizes between *d* = 0.58 and *d* = 0.71 in SEBR, and between *d* = 0.91 and *d* = 1.02 in pupil dilation. Even based on the second study with higher effect size, a fear reduction of 50% in SEBR in the doxycycline group would correspond to an effect size of *d* = 0.46. Post hoc with *N* = 100, power to detect this difference at an alpha rate of 0.05 in a one-tailed *t*-test is 74%. We note that our primary pre-registered analysis is a linear mixed effects (LME) model, not a *t*-test; however, effect size estimation with LME is not well established and thus the power analysis was based on a *t*-test.

### Study medication

The tetracyclic antibiotic doxycycline (brand name: Vibramycin; Pfizer, Zurich Switzerland) was used as study medication, mannitol as placebo. Doxycycline (50 μM) completely inhibits phorbol-12-myristate-13-acetate (PMA)-mediated induction of MMP-9 measured by Western blotting and gelatin zymography (Hanemaaijer et al. 1998). This inhibition can also be observed at the mRNA level (Hanemaaijer et al. 1998). An in vitro study by Modheji et al., (2016) investigated the inhibitory effects of doxycycline monohydrate on MMP-9 activity using zymography. Complete inhibition did not occur with the dose of 500 µM of doxycycline; however, total MMP-9 activity inhibition was seen with 4 mM doxycycline. The study dose of 200 mg orally was based on a previous study using delay fear conditioning (Bach et al. 2018). Clinically, doxycycline is used to treat neuroborreliosis (Dotevall and Hagberg 1989) based on its ability to penetrate the blood brain barrier (Mento et al. 1969). Doxycycline is detectable in cerebrospinal fluid beginning 2–4 h after ingestion (Dotevall and Hagberg 1989; Karlsson et al. 1996). Thus, and for consistency with previous studies (Bach et al. 2018, 2019; Wehrli et al. 2023), fear memory acquisition was scheduled ~ 3.5 h after drug ingestion.

As the drug’s half-life is approximately 16 h, the drug would have been cleared by 99.9% after 7 days on visit 3. A GMP-licensed pharmacy (Kantonsapotheke, Zurich Switzerland) manufactured, blinded, and randomised the drug, separately for males and females. Randomisation was not unblinded until the last participant completed the study, data were checked for consistency and the analysis plan was pre-registered on OSF (https://osf.io/4xtm2/

### Stimuli

**Fig. 2:**
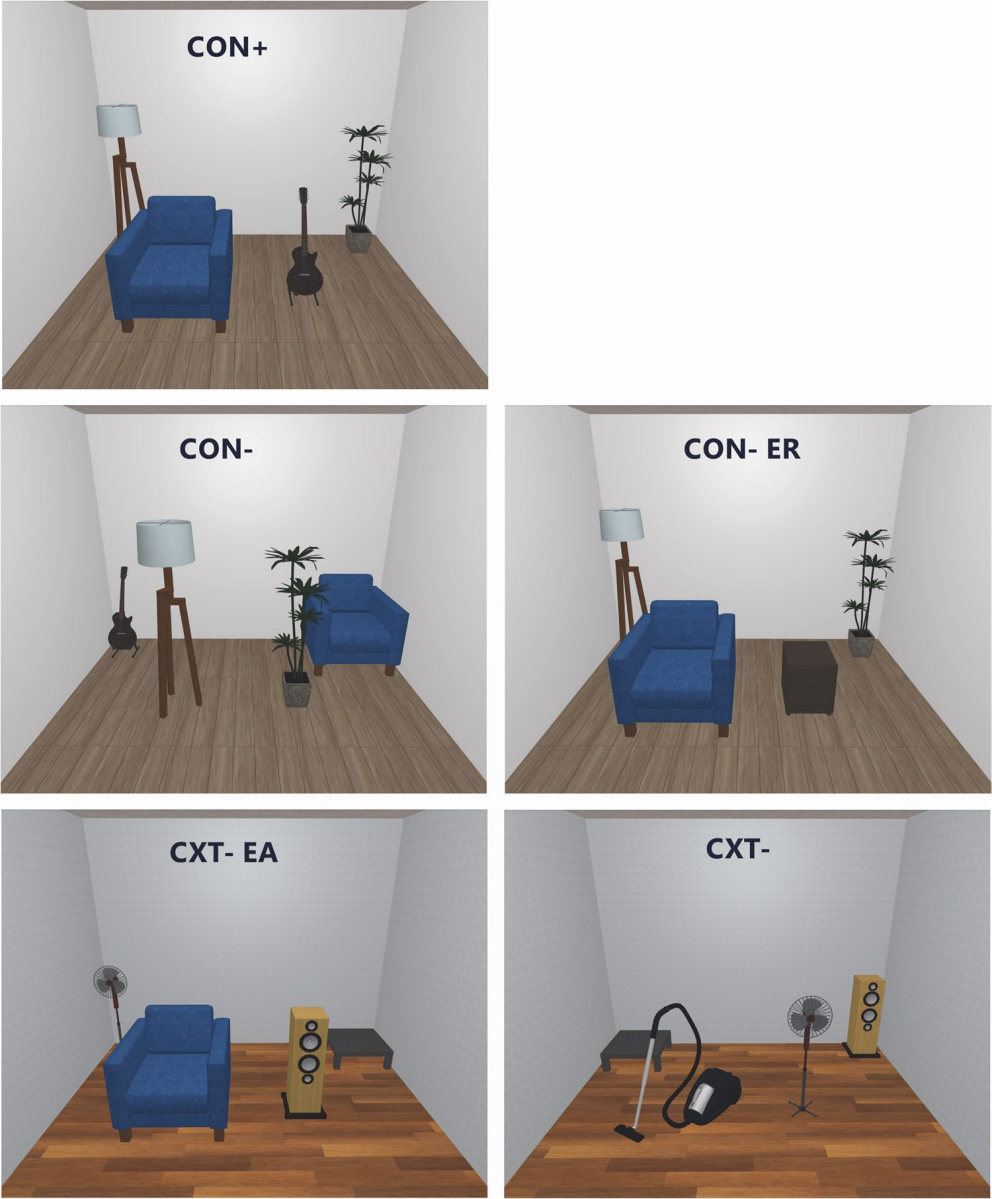
Static room pictures of configural conditioning. Configural CS + (CON +). Configural CS- have the same items as CON + but rearranged (CON-), or one item replaced (CON-ER). New context CS- have different walls and floor than CON + . One new context CS- has one element added from CON + (CXT-EA), the other has all new items (CXT-)

A configural fear conditioning paradigm was adapted from Stout et al. (2018, 2019) and employed five static room pictures as experimental contexts (see Fig. 2). The room pictures are composed of five walls (including ceiling and floor) and four furniture items. Only one room was associated with the unconditioned stimulus (US): Configural CS + (CON +). The other four rooms (CS- rooms) differ gradually from the CON + room. Two rooms have the same walls as the CON + room, but their furniture is rearranged (configural CS-; CON-) or one item is replaced (configural CS- element replaced; CON- ER). Two more rooms have different walls than the CON ± rooms, one room contains one furniture item from CON + in the same position, and three new items (new context CS- element added; CXT-EA). The other contains only new items (new context CS-; CXT-). To control for room similarity, CS + /US association was the same for all participants. Participants were not instructed about the association, but were informed that one of the rooms would be most predictive of US. For the analysis, CS + (CON +) was contrasted both with all CS- conditions individually (CS-_1,2,3,4_), or with the average response to the CS- conditions (CS-_ave_). The configural task was presented on a 20″ screen (Dell P2014h), set to an aspect ratio of 4:3 at 60 Hz, with a resolution of 1280 × 1024. To minimize head movement, participants were placed in a headrest, positioned at 70 cm distance from the screen and 47 cm from the eye-tracker. The experiment was conducted in a dark, sound-proof chamber.

Before acquisition training started, participants were familiarised with the room pictures, by showing them first simultaneously and then consecutively in random order, until all rooms had been shown twice. In acquisition training, participants were presented with 88 trials in four blocks. Each block consisted of six CS + (CON +) trials and four trials of each CS- condition (CON-, CON- ER, CXT-, CXT- EA), in total 24 CS + / 16 each CS-. Trials were pseudo-randomly ordered, with the first ten trials of each block consisting of two presentations of the five room images in random order. The remaining 12 trials (four CS + trials and two of each CS- image) where then presented in random order. All room pictures were presented for 7.5 s in full screen mode. US was presented 7.0 s after trials onset, for 83% of CS + trials. US always co-terminated with CS + presentation. None of the CS- conditions were reinforced. Inter trial intervals (ITI) were jittered between 7.5 – 10.5 s and consisted of the presentation of a light grey background (RGB: 178.5, 178.5, 178.5) on the computer screen. Participants were asked to respond to each CS presentation by pressing the “down” arrow key as soon as they recognized the room configuration, irrespective of shock association. In the recall test, 88 trials were presented with the same logic of order as in the acquisition training. No US were delivered in the recall test, but each CS presentation co-terminated with a startle probe, which was presented 7.0 s after trial onset. The re-learning session was structured similarly to acquisition training, but consisted of only 44 trials, balanced into two blocks.

US was an electric shock of 500 ms duration, consisting of a sequence of 250 square electric pulses with a 10% duty cycle. Shocks were generated by a constant current simulator (Digitimer DS7A, Digitimer, Welwyn Garden City UK) and delivered to the participants dominant forearm with a pin-cathode/ring anode configuration. Calibration of shock intensity followed the same procedure as our previous studies (Bach et al. 2018, 2019; Wehrli et al. 2022); US intensity was set to 80–90% of the lowest painful stimulus. Startle probe was a 40 ms white noise burst of approximately 102 dB loudness and instantaneous rise time, delivered binaurally with headphones (HD, 202 Sennheiser, Wedemark-Wennebostel, Germany).

After each session, participants were asked to rate CS-US contingency (0 = never received a shock, 100 = always received a shock) as well as their arousal (0 = very calm, 100 = very excited) and valence (0 = very unhappy, 100 = very happy) to each room picture on a 0–100% continuous scale. After the recall test, participants were additionally asked to recall the contingency of the acquisition training for each CS.

### Procedure

#### Screening visit 1 (day -14 to day -2)

Study procedure is illustrated in Fig. 1b. To verify inclusion criteria, participants were medically screened on visit 1 by the study physician. Blood and urine samples were collected and analysed by a medical laboratory to control participant’s health status, exclude pregnancy and test for drug use. Participants were screened for depression with Beck’s Depression Inventory (BDI-II) (Beck, 1996). Participants who scored above 14 points, indicating mild depressive symptoms, were not enrolled into the study. Furthermore, individual US intensity was calibrated, and startle sounds were presented to verify participants' tolerance of these sounds.

#### Acquisition visit 2 (day 0)

Acquisition visit 2 always started in the morning, between 07:45 and 10:30 am. Participants were asked about their health status, medication, and drug consumption since visit 1. Afterwards, participants orally ingested the study drug and were monitored by study staff during a metabolization period lasting approximately 180 min. In the hour before and after drug ingestion, participants were asked to refrain from eating or drinking beverages containing milk, as this might influence the absorption of doxycycline (Meyer et al. 1989). After the metabolization period, participants filled in the State-Trait Anxiety Inventory (STAI) (Laux et al. 1981), and US intensity was calibrated again. Subsequently, the fear acquisition paradigm started around 210 min after drug intake. Following acquisition, US calibration stimuli were presented again to control for US habituation or sensitization.

#### Recall and re-learning visit 3 (day + 7)

Recall visit 3 took place exactly 7 days after the acquisition visit 2. Participants were seated in the same experimental room as in visit 2, the shock electrode was attached in the same position as in visit 2, and participants were instructed that they might receive a US. Startle probes were presented 8 times for habituation. During the subsequent recall test, none of the CS were reinforced. Startle probes were presented on all trials. After recall test, participants were again asked to rate CS-US contingency and their recollection of the shock association in visit 2. Immediately afterwards, the re-learning session followed, with the same US intensity as in acquisition. No startle probes were presented in this session.

### Psychophysiological recordings

To record electromyogram (EMG), two 4 mm AG/AgCl cup electrodes with high conductance gel were positioned on the orbicularis oculi muscle of the participants’ left eye, one on the lower eyelid in a vertical line to the pupil in a forward gaze, the other beneath the lateral canthus at ca. 1–2 cm interelectrode distance (Blumenthal et al. 2005). Electromyogram was amplified with a gain of 2000, and band-pass filtered at 1–500 Hz (EMG100C, Biopac Systems). For skin conductance recording, two disposable Ag/AgCl snap electrodes (EL507, Biopac Systems), filled with 0.5% NaCl electrolyte gel (Hygge & Hugdahl 1985) (GEL101, Biopac Systems) were placed on the thenar/hypothenar of the participants non-dominant hand, and an additional ground electrode (FS-TC1, Skintact / EL503, Biopac Systems) was placed on the non-dominant elbow. Skin conductance was measured with a 0.5 V constant voltage (EDA100C, Biopac Systems). Both EMG and skin conductance signals were digitized at 2000 Hz (MP160, Biopac Systems) and recorded (Acknowledge, Biopac Systems).

Pupil diameter and gaze direction were recorded with an EyeLink 1000 System (SR Research) with a 500 Hz sampling rate. To calibrate gaze direction, we used the standard nine-point protocol implemented in the EyeLink 1000 Software.

### Data analysis

We pre-registered our analysis plan on OSF (https://osf.io/4xtm2/) before unblinding the drug randomization. Pre-processing, modelling and analysis of psychophysiological data was done using MATLAB (Version 2018b, Math Works), with procedures implemented in the PsPM toolbox version 5.1.1 (Psychophysiological modelling, bachlab.github.io/pspm) and R 4.0.2 (www.r-project.org). Analysis code is available on OSF (https://osf.io/4xtm2/). The anonymized dataset is available on Zenodo (https://zenodo.org/records/7601734).

#### Conditioned response scoring

Prior methodological work (Xia et al. 2023) with the same experimental paradigm identified SEBR and pupil dilation as indices for configural fear memory retention 7 days after fear learning, and skin conductance response (SCR) and pupil dilation as indices for fear acquisition. Pre-processing and scoring methods were in line with the most sensitive procedures identified in this previous work. To control for data quality, response estimates for each trial and participant were extracted. For each trial, data points outside of three standard deviations around the group mean were excluded. This removed fewer than 1.5% of trials. For condition-wise analysis, data of each participant outside three standard deviations of the corresponding group mean was excluded. This excluded six participants for SCR and three for pupil dilation in acquisition training, as well as five for SCR and four for pupil dilation in the re-learning session.

### Data pre-processing and model-based analysis

#### Startle-eye blink responses

EMG timeseries data was filtered with a fourth order Butterworth filter with a cut-off of 50–470 Hz, and a notch filter of 50 Hz to remove mains noise. Next, data were rectified and smoothed with a fourth order Butterworth low-pass filter and a 3 ms time constant. Pre-processed EMG data was then visually inspected. Two participants with no discernible SEBR in most trials were excluded from the EMG analysis. SEBR amplitudes were estimated using a GLM with canonical response function and flexible latency within a 0.15 s window from startle onset, identified from the audio recording using PsPM procedures (Khemka et al. 2017). Four participants had technical failures in the recording of startle sounds; for these participants the onset of startle presentation was estimated to be 7 s after CS onset. Trial-wise SEBR estimates were then normalised for each participant by their CS-_ave_ response.

#### Skin conductance responses

Skin conductance was visually inspected to identify participants without unconditioned response; this resulted in no exclusions. Data was then filtered with a first-order bidirectional band-pass Butterworth filter (cut-off frequencies: 0.0159–5 Hz) and down sampled to 10 Hz (Staib et al. 2015). To estimate SCR amplitudes, a non-linear psychophysiological model in PsPM was implemented (Bach et al. 2010; Gerster et al. 2018; Staib et al. 2015), modelling a fixed-latency SCR to CS onset. Trial-wise SCR amplitude estimates were then normalised for each participant by their CS-_ave_ response.

#### Pupil dilation

Pupil data was converted from arbitrary units to millimetres with standard procedures in PsPM. Pre-processing followed the procedure by Kret and Sjak-Shie (2019) as implemented in PsPM. Foreshortening error correction was performed (Hayes and Petrov 2016), and data points with gaze coordinates outside the visual region of interest (i.e., exceeding ± 9.27° visual angle) were excluded. To correct for luminance differences between the five CS images inducing pupil size changes, we created a luminance time series from the image presentation (Korn and Bach 2016) and included these into the standard GLM approach in PsPM for fear conditioning (Korn et al. 2017; Korn and Bach 2016) as nuisance regressor. One participant was excluded from analysis because the eye-tracker had been inadvertently moved. Furthermore, participants with more than 50% missing data during CS presentation (CS presentation onset—offset) were excluded. This excluded four participants in acquisition, one in the recall test, and five in the re-learning session. Additionally, individual trials with more than 50% missing data were excluded.

### Statistical analysis

#### Acquisition and re-learning session

Following the pre-registration for our primary analysis, amplitude estimates of non-reinforced trials were averaged for each condition, both for SCR and pupil dilation. Group differences between CS + and the CS-_ave_ trials were compared in a paired *t*-test, for both the placebo and doxycycline group, and between groups. As a secondary analysis, we performed a linear mixed effect (LME) model analysis using lmer() of the “lme4” package version 1.1.23, accounting for simple and interaction effects of 2 × (drug) × 5 (CS + / CS-_1,2,3,4_) × 88 (trials) in acquisition, and 2 × (drug) × 5 (CS + / CS-_1,2,3,4_) × 44 (trials) in re-learning, with random intercept (formula: lmer(data ~ drug*conditions*trial index + (1|subject)). Mean-centred trial number was used as a linear indicator of time. As there is a possibility of US bias, only non-reinforced trials were entered into the LME analysis and reinforced trials were treated as missing data. Pupil dilation responses are shorter than the CS duration (Korn et al. 2017; Korn and Bach 2016), and as such the US bias might be negligible. A robustness analysis including all trials for pupil dilation largely confirmed these results (for details see SI Table S10).

#### Recall test

Our primary pre-registered analysis for both SEBR estimates, and pupil dilation was an LME with 2 x (drug) × 5 (CS + /CS-_1,2,3,4_) × 88 (trials) with random intercept (formula: data ~ drug*conditions*trial index + (1|subject)). As a secondary analysis, we performed a paired t-test for the CS + /CS-_ave_ differences within and between the groups. Because position of trials in CS + and CS- are only perfectly balanced over the first 10 trials, trials were sorted into four subsets per block. The first two subsets entail the first or second trial of each condition, respectively, therefore covering the first ten balanced trials. We performed paired-tests comparing CS + and CS-_ave_ trials for the first and the average of the first two subsets for the placebo and doxycycline group, and then compared the difference of CS + /CS-_ave_ between the placebo and doxycycline group.

## Results

### Configural fear acquisition

#### Pupil dilation

**Fig. 3:**
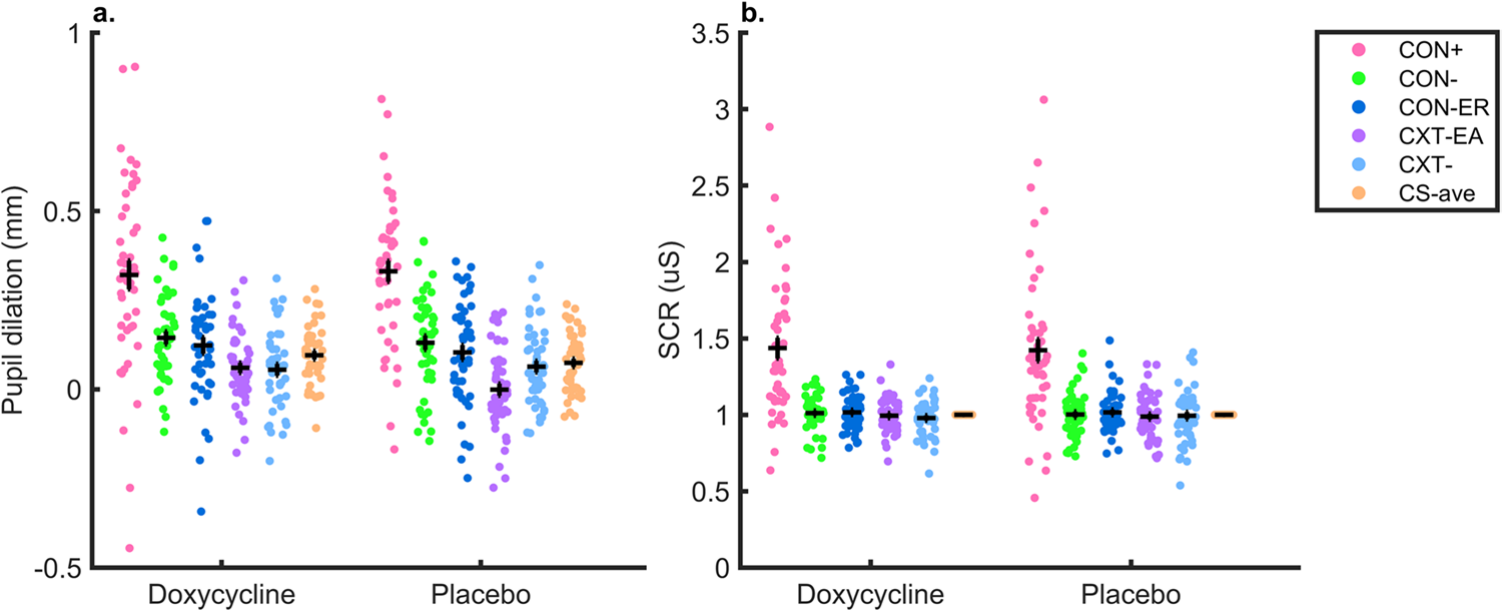
Memory acquisition, for CS + /US-, CS-_1,2,3,4_ and CS- average. **a**: Pupil dilation, **b**: Normalised SCR to CS onset in memory acquisition phase. Only data of non-reinforced trials are included. CS-_ave_ corresponds to the averaged data over the 4 CS- conditions. Black cross depicts group mean ± SEM

First, we sought to verify configural fear learning in the placebo group. Averaged over the entire acquisition session, we found CS + /CS-_ave_ differentiation for pupil dilation both in the placebo *(t(61)* = *8.08, p* < 0.001*, d = *1.18, g* = *1.16)* and doxycycline group *(t(52.44)* = *5.29, p* < 0.001*, d = *0.79, g* = *0.78),* indicating successful learning of the CS-US association in both groups (see Fig. 3). A group comparison revealed no significant difference *(t(90)* = *-0.68, p* = *0.50, d* = *-0.14, g* = *-0.14)* (see SI Table S2 and S3). As a secondary analysis, we investigated the group differences in a pre-registered LME model. This revealed differential learning (main effect CS), habituation (main effect trial) and a CS x drug interaction, with stronger learning in the placebo group (see Table 1).

**Table 1:** LME analysis of pupil dilation and skin conductance during fear acquisition and re-learning, as well as startle eye-blink responses and pupil dilation during recall. Significant effects are marked with “*”

Fear acquisition	Pupil dilation				Skin Conductance			
Effect	*F-value*	*df*		*p-value*	*F-value*	*df*		*p-value*
Drug (doxycycline/placebo)	0.10	1,	6346	0.75	0.00	1,	6681	0.98
Trial number	171.02	1,	6346	< .001*	543.77	1,	6681	< .001*
Condition (CS + /CS-_1,2,3,4_)	65.67	4,	6346	< .001*	19.59	4,	6681	< .001*
Drug x Trial	0.03	1,	6346	0.86	4.78	1,	6681	0.029*
Drug x Condition	2.63	4,	6346	0.033*	0.09	4,	6681	0.99
Trial x Condition	1.06	4,	6346	0.37	2.74	4,	6681	0.027*
Drug x Trial x Condition	1.62	4,	6346	0.17	0.53	4,	6681	0.71
Fear recall	Startle Eye-blink				Pupil dilation			
Effect	*F-value*	*df*		*p-value*	*F-value*	*df*		*p-value*
Drug (doxycycline/placebo)	0.55	1,	8507	0.46	1.58	1,	8507	0.201
Trial number	1996.41	1,	8507	< .001*	158.64	1,	8507	< .001*
Condition (CS + /CS-_1,2,3,4_)	101.56	4,	8507	< .001*	57.00	4,	8507	< .001*
Drug x Trial	0.08	1,	8507	0.78	2.91	1,	8507	0.09
Drug x Condition	1.56	4,	8507	0.18	0.66	4,	8507	0.62
Trial x Condition	6.64	4,	8507	< .001*	14.51	4,	8507	< .001*
Drug x Trial x Condition	2.57	4,	8507	0.036*	0.29	4,	8507	0.89
Fear re-learning	Pupil dilation				Skin Conductance			
Effect	*F-value*	*df*		*p-value*	*F-value*	*df*		*p-value*
Drug (doxycycline/placebo)	1.10	1,	2984	0.30	0.60	1,	3182	0.44
Trial number	13.54	1,	2984	< .001*	244.93	1,	3182	< .001*
Condition (CS + /CS-_1,2,3,4_)	62.33	4,	2984	< .001*	21.13	4,	3182	< .001*
Drug x Trial	3.50	1,	2984	0.062	16.31	1,	3182	< .001*
Drug x Condition	0.31	4,	2984	0.87	2.39	4,	3182	0.048*
Trial x Condition	2.44	4,	2984	0.045*	6.36	4,	3182	< .001*
Drug x Trial x Condition	0.31	4,	2984	0.87	1.03	4,	3182	0.39

#### Skin conductance responses

Analysis of SCR data confirmed successful CS + / CS-_ave_ differentiation both for the placebo *(t(48)* = *5.82, p* < 0.001*, d = *0.83, g* = *0.82)* and the doxycycline group *(t(44)* = *6.43, p* < 0.001*, d = *0.96, g* = *0.94)*, when averaged over the entire session (see Fig. 3). We found no group difference between placebo and doxycycline group *(t(92)* = *0.14, p* = *0.89, d* = *0.03, g* = *0.03)* (see SI Table S2 and S3). The LME model confirmed habituation and differential learning, as well as more habituation in the placebo group and for CS CON + (see Table 1).

### Configural fear memory recall

#### Startle eye-blink responses

Next, we sought to confirm contextual fear memory retention a week after fear acquisition in the placebo group. We found differential memory recall (main effect CS) *(F(4, 4341)* = *45.35, p* < 0.001*), habituation (main effect trial number) *(F(1, 4341)* = *1041.75, p* < 0.001*), and no CS x trial interaction *(F(4, 4341)* = *0.73, p* = *0.57).*

**Fig. 4:**
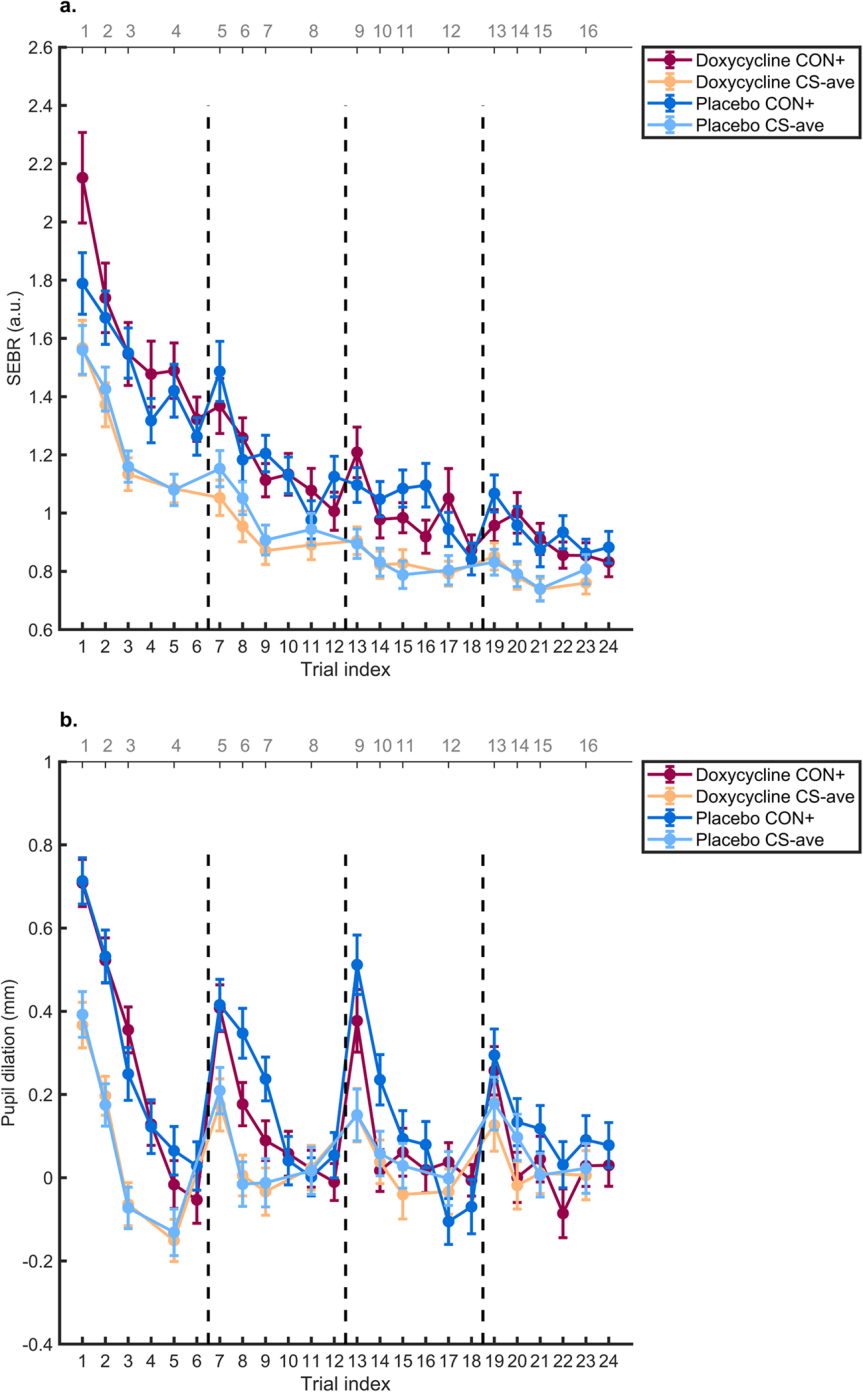
Memory recall, time course for CS + and CS-_ave_ ± SEM. **a**: normalised SEBR, **b**: Pupil dilation. Dots depict group mean, vertical lines depict SEM

Our primary analysis was the comparison of doxycycline and placebo in an LME model. This confirmed differential memory recall (main effect CS), and habituation (main effect trial number), across both groups, as well as more habituation for CON + , in the doxycycline group. However, there was no evidence that doxycycline had an impact on contextual fear memory retention across the entire recall test (see Table 1 and Fig. 4). Instead, differential fear recall was increased under doxycycline (rather than decreased, as hypothesized) in early trials (interaction drug x trial x condition).

**Fig. 5:**
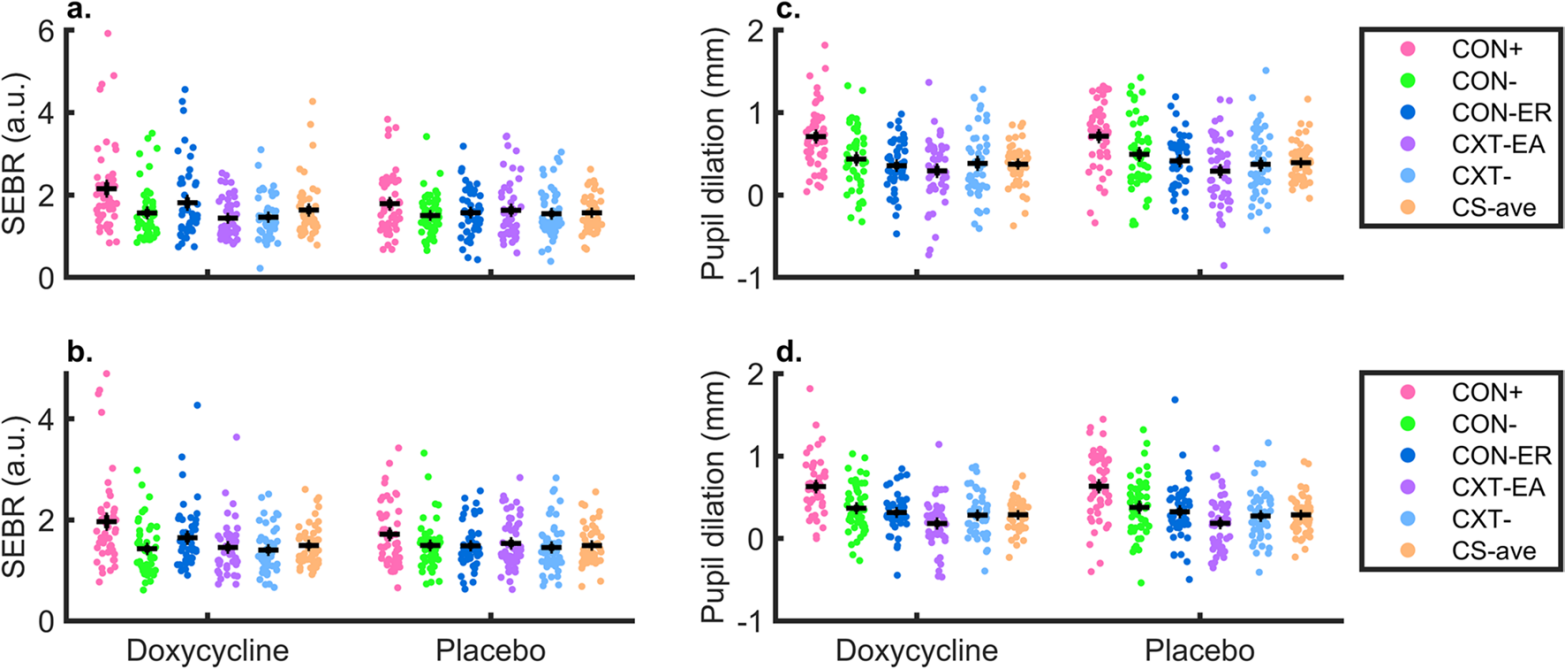
Memory recall, for CS + , CS-_1,2,3,4_ and CS-_ave_. **a**: normalised SEBR first subset, **b**: normalised SEBR first two subsets, **c**: Pupil dilation first subset, **d**: Pupil dilation first two subsets. Black cross depicts group mean ± SEM

When looking into the first and first two subsets of the retention session for the placebo group, we found a significant CS + / CS-_ave_ differentiation in the placebo group in the first *(t(47)* = *2.62, p* = 0.012*, d = *0.37, g* = *0.36)* and the average of the first two subsets *(t(49)* = *3.39, p* = 0.001*, d = *0.48, g* = *0.47)*. We also found successful differentiation in the doxycycline group in the first *(t(46)* = *2.52, p* = 0.015*, d = *0.36, g* = *0.36)* and the average of the first two subsets *(t(47)* = *2.54, p* = 0.014*, d = *0.37, g* = *0.36)* (see Fig. 5 and SI Table S4). When comparing the groups, we found a trend-level effect *(t(93)* = *1.95, p* = *0.054, d* = *0.39, g* = *0.39)* in the first subset*,* with the doxycycline group showing larger differences between CS + and CS-_ave_ trials, i.e., the opposite of what we had hypothesised. We found no difference when averaged over the first two subsets *(t(96)* = *1.69, p* = *0.093, d* = *0.34, g* = *0.34)* (see SI Table S5).

#### Pupil dilation

In pupil dilation, we found no drug effect in the LME, but effects of trial, CS, and an interaction effect of trial x CS (see Table 1 and Fig. 4). This results align with t-tests, showing significant differences between CS + /CS-_ave_ in the first subset *(t(48)* = *5.41, p* < 0.001*, d = *0.76, g* = *0.75)* and the average of the first two subsets *(t(50)* = *6.34, p* < 0.001*, d = *0.89, g* = *0.87)* of the placebo group, as well as in the doxycycline group; first subset *(t(46)* = *5.83, p* < 0.001*, d = *0.85, g* = *0.84)* first two subsets *(t(46)* = *6.58, p* < 0.001*, d = *0.96, g* = *0.94)* (see Fig. 5 and SI Table S6). We found no difference between the placebo and doxycycline group in the first *(t(94)* = *0.20, p* = *0.84, d* = *0.04, g* = *0.04)* and the average of the first two subsets *(t(96)* = *-0.06, p* = *0.95, d* = *-0.01, g* = *-0.01)* (see SI Table S7).

### Configural re-learning

#### Pupil dilation

For re-learning, we performed the same analysis as for the acquisition. When averaged over the entire session and all CS- types, we found a significant difference for CS + /

CS-_ave_ in the placebo *(t(55)* = *4.47, p* < 0.001*, d = *0.67, g* = *0.66)* and doxycycline group *(t(54.71)* = *4.53, p* < 0.001*, d = *0.69, g* = *0.68)* for pupil dilation (see SI Table S8), and no group difference *(t(85)* = *0.03, p* = *0.98, d* = *0.01, g* = *0.01)* (see SI Table S9). In the LME we found an effect of trial, CS, and an interaction trial x CS, indicating stronger habituation for CON + (see Table 1).

#### Skin conductance responses

In SCR, we again found CS + / CS-_ave_ differentiation in re-learning both for the placebo *(t(47)* = *5.00, p* < 0.001*, d = *0.72, g* = *0.71)* and doxycycline group *(t(43)* = *4.00, p* < 0.001*, d = *0.60, g* = *0.59)* (see SI Table S8). We found no group difference *(t(90)* = *-1.70, p* = *0.09, d* = *-0.35, g* = *-0.34)* (see SI Table S9). Similar as in acquisition, we found effects of trial and CS, as well as interactions for, group x CS (stronger responses in the placebo group), group x trial (placebo group habituates quicker) and trial x CS (CON + shows faster decline than the other conditions) in the LME (see Table 1).

#### Contingency memory

Using a three-way mixed ANOVA, we tested for effects on subjective contingency ratings. We found that participants differentiated the CS-rooms in their subjective ratings (main effect CS, *p* < 0.001*) and that the average ratings changed over time (main effect time point, *p* < 0.001*). Furthermore, participants updated contingency memory after recall and again after re-learning (interaction CS x time point, *p* < 0.001*). We found no difference between doxycycline and placebo group (see Table 2 for details).

**Table 2:** Three-way mixed ANOVA (1 between: drug and 2-within subject factors: timepoint and condition) of contingency ratings, arousal and valence. Significant effects are marked with “*”

	Contingency			Arousal			Valence		
Effect	*F-value*	*df*	*p-value*	*F-value*	*df*	*p-value*	*F-value*	*df*	*p-value*
Drug (doxycycline/placebo)	0.23	1, 96	0.63	1.61	1, 96	0.21	0.42	1, 96	0.52
Timepoint	259.84	3, 288	< .001*	1.69	2, 192	1.87	1.19	2, 192	0.31
Condition (CS + /CS-_1,2,3,4_)	2422.48	4, 384	< .001*	309.66	4, 384	< .001*	114.88	4, 384	< .001*
Drug x Timepoint	0.42	3, 288	0.74	0.65	2, 192	0.52	0.86	2, 192	0.42
Drug x Condition	1.37	4, 384	0.24	0.99	4, 384	0.41	1.93	4, 384	0.11
Timepoint x Condition	382.98	12, 1152	< .001*	22.09	8, 768	0.19	8.69	8, 768	< .001*
Drug x Timepoint x Condition	0.99	12, 1152	0.46	0.14	8, 768	1.00	0.83	8, 768	0.58

## Discussion

In the present work, we investigated the effect of doxycycline on configural fear memory recall. We found successful fear memory recall in our registered primary (SEBR) and secondary (pupil dilation) outcome, both for the placebo and the doxycycline group. However, we observed no reduction in fear recall in the doxycycline group. On the contrary, it appeared that learning was slightly (but non-significantly) stronger in the doxycycline group. Secondary analyses revealed no indication of impaired fear memory consolidation in the doxycycline group.

There was no evidence that our negative result could be due to a lack of learning in the placebo group. Effect sizes for learning in the placebo group were around *d* = 1.3. A secondary analysis of pupil responses revealed some evidence for stronger learning in the placebo group. However, this would not mask an impact of doxycycline on consolidation, because our main analysis was a direct group comparison within the recall test which would be increased by a group difference during acquisition.

There was also no evidence either that our negative result could be due to a lack of memory retention in the placebo group. Indeed, both groups showed significant memory retention when analysed separately. We do note, however, that effect sizes for SEBR in the placebo group (around *d* = 0.40) were smaller than those assumed during study planning (*d* = 1.17) or in the preceding methodological study (*d* = 0.70), such that power to detect group differences was smaller than anticipated. Generally, across several studies we observed effect sizes in a placebo group to be smaller than in preceding methodological work, potentially due to the increased complexity of the procedure in a drug study or due to sample bias. This would motivate using larger samples for future studies.

Our finding qualifies previous work on the impact of doxycycline on fear memory consolidation in important ways. While a previous study (Bach et al. 2018) suggested that doxycycline impairs cue fear memory recall, Wehrli et al. (2023) only found weak evidence in favour of a doxycycline influence on trace fear recall. In contrast, the current study finds no evidence for an inhibition of context fear recall, and in fact some evidence to the contrary. What could account for this discrepancy? First, previous results might simply constitute false positives, and in this case, doxycycline might not have an impact on any form of human fear conditioning. As an underlying reason, either MMP-9 might not be crucial for fear memory consolidation, or doxycycline might not sufficiently inhibit MMP-9. A rodent study (Brown et al. 2009) showed no impact of a selective MMP-9 inhibitor on fear conditioning, although this experiment did find an impact of MMP-9 inhibition on fear memory reconsolidation. As a second possibility, doxycycline might have an impact on cued (and possibly trace) fear consolidation but not on configural fear consolidation with putatively different neural circuits involved. Although previous rodent work has demonstrated impaired spatial learning under MMP-9 inhibition (Wright et al. 2007), our configural fear learning paradigm involves circuits that go beyond those involved in spatial learning (e.g. hippocampus-amygdala projections (Castegnetti et al. 2021)). Also, doxycycline is likely to influence MMPs other than MMP-9 (Burggraf et al. 2007; Golub et al. 1991) which might also influence synaptic reconfiguration at the same or other synapses (Huntley 2012). We note that the initial cue fear conditioning study by Bach et al. (2018) has so far not been directly or conceptually replicated. As a way forward to disambiguate between the different interpretations of the current work, we suggest a direct replication of Bach et al. (2018). It might also be useful to consider drugs that are more specific inhibitors of MMP-9 such as minocycline (Cunha 2000; Modheji et al. 2016), or more potent application methods of doxycycline, e.g., via injection.

Future studies could also seek to disambiguate the mnemonic process targeted. In the present and previous work, doxycycline was given before memory encoding because of its slow uptake. This meant that it could have impacted on acquisition and/or consolidation. In any secondary prevention setting however, an intervention would be applied after memory encoding, and would thus be restricted to impacting consolidation. To test whether a drug specifically impacts on consolidation and is feasible for clinical application by administering it after memory encoding would require that uptake into the CNS is faster than the targeted consolidation process. This might be achieved by using minocycline and/or generally by drug injection.

Based on the current results, it seems plausible that a single dose of doxycycline (of 200 mg) is not appropriate to significantly alter fear memory consolidation in complex and realistic tasks beyond cued fear conditioning. This should, for the moment, limit translation to clinical populations. Further investigations would be needed to replicate previous results. However, given limited potential for clinical application of doxycycline, it might be a better use of resources to investigate alternative drugs in the current model.

### Supplementary Information

Below is the link to the electronic supplementary material.

Supplementary file1 (PDF 195 KB)
