# Supplementary material for: The impact of doxycycline on human contextual fear memory
Source: Psychopharmacology (Berl). 2024 Feb 9;241(5):1065–77. doi: 10.1007/s00213-024-06540-w (PMC11031495; doi:10.1007/s00213-024-06540-w)
Supplement: Supplementary file 1 — Supplementary file1 (PDF 195 KB) [file 213_2024_6540_MOESM1_ESM.pdf]

The impact of doxycycline on human contextual fear memory
Wehrli, J., Xia, Y., Abivardi, A., Kleim, B. Bach D. - 2024

Table S1

Sample characteristics: US intensity: Electric current used in experiment; Pain ratings pre- vs. post: Difference in average pain ratings of 14 stimuli before and after the acquisition test; State Anxiety: Measured with State-Trait Anxiety Inventory (STAI) (Laux et al., 1981); Trait Anxiety: Measured with State-Trait Anxie-ty Inventory (STAI) (Laux et al., 1981); BDI: Beck Depression Inventory (Beck, 1996); P-value: two-sample t-test; Cohen's d: Cohen's d effect size of the group difference.

	Placebo		***Doxycycline***			
Sex	***Male (n = 26)***	***Female (n = 26)***	***Male (n = 24)***	***Female (n = 24)***		
	mean	***s.d.***	***mean***	***s.d.***	***P-value***	***Cohen's d***
Age (years)	23.69	3.60	23.92	3.99	0.77	0.06
Weight (kg)	68.50	10.15	69.34	13.89	0.73	0.07
US intensity (mA)	4.35	2.15	4.27	1.58	0.83	0.04
Pain rating pre- vs. post	12.10	14.52	9.55	17.81	0.43	0.16
State Anxiety pre acquisition	32.44	7.04	31.35	6.36	0.42	0.16
State Anxiety pre recall	31.17	6.69	30.58	6.85	0.66	0.09
Trait Anxiety pre acquisition	31.90	7.70	30.96	5.76	0.49	0.14
BDI screening	1.63	1.93	1.73	1.92	0.81	0.05
BDI post recall	1.79	3.04	2.63	3.40	0.20	0.26
Temperature acquisition (°C)	21.89	1.24	22.14	0.95	0.25	0.23
Temperature recall (°C)	22.06	1.16	22.11	1.08	0.83	0.04
Humidity acquisition (%)	48.89	10.05	49.18	8.55	0.88	0.03
Humidity recall (%)	49.41	10.02	50.29	7.96	0.63	0.10
Metabolization time (min)	208.52	14.55	210.69	9.65	0.38	0.17

Table S2
Acquisition paired t-test CS+/CS- (CS- 1,2,3,4 and CS- average), not corrected for multiple comparisons

Measure	Group	subsets	Conditions	t-statistic	p	df	95% CI	cohen's d	hedge's g
SCR DCM	Placebo	all	CON+ vs. CON-	5.57	<.001*	55.98	[0.27, 0.57]	0.80	0.78
		"	CON+ vs. CON-ER	5.43	<.001*	54.45	[0.26, 0.56]	0.78	0.76
		"	CON+ vs. CXT-EA	5.76	<.001*	55.66	[0.28, 0.59]	0.82	0.81
		"	CON+ vs. CXT-	5.59	<.001*	58.93	[0.28, 0.58]	0.80	0.79
		"	CON+ vs. CS-ave.	5.82	<.001*	48	[0.28, 0.57]	0.83	0.82
	Doxycycline	all	CON+ vs. CON-	6.08	<.001*	49	[0.29, 0.57]	0.91	0.89
		"	CON+ vs. CON-ER	6.00	<.001*	50	[0.28, 0.56]	0.89	0.88
		"	CON+ vs. CXT-EA	6.31	<.001*	50	[0.30, 0.58]	0.94	0.92
		"	CON+ vs. CXT-	6.51	<.001*	50	[0.32, 0.60]	0.97	0.95
		"	CON+ vs. CS-ave.	6.43	<.001*	44	[0.30, 0.57]	0.96	0.94
Pupil dilation	Placebo	all	CON+ vs. CON-	5.60	<.001*	82	[0.13, 0.27]	0.82	0.80
		"	CON+ vs. CON-ER	6.27	<.001*	84	[0.16, 0.30]	0.91	0.90
		"	CON+ vs. CXT-EA	9.76	<.001*	74	[0.26, 0.40]	1.42	1.40
		"	CON+ vs. CXT-	7.96	<.001*	72	[0.20, 0.33]	1.16	1.14
		"	CON+ vs. CS-ave.	8.08	<.001*	61	[0.19, 0.32]	1.18	1.16
	Doxycycline	all	CON+ vs. CON-	3.97	<.001*	60.82	[0.09, 0.27]	0.59	0.58
		"	CON+ vs. CON-ER	4.21	<.001*	70.57	[0.10, 0.29]	0.63	0.62
		"	CON+ vs. CXT-EA	6.04	<.001*	55.09	[0.17, 0.35]	0.90	0.88
		"	CON+ vs. CXT-	6.05	<.001*	58.57	[0.18, 0.35]	0.90	0.89
		"	CON+ vs. CS-ave.	5.29	<.001*	52.44	[0.14, 0.31]	0.79	0.78

Table S3
Acquisition independent t-test between CS+/CS- (CS- 1,2,3,4 and CS- average),
difference for placebo and doxycycline group, not corrected for multiple comparisons

Measure	Group	subsets	Conditions	t-statistic	p	df	95% CI	cohen's d	hedge's g
SCR	Placebo vs. Doxycycline	all	CON+ vs. CON-	0.05	0.96	90.72	[-0.20, 0.21]	0.01	0.01
		"	CON+ vs. CON-ER	0.14	0.89	91.92	[-0.19, 0.22]	0.03	0.03
		"	CON+ vs. CXT-EA	0.08	0.94	91.59	[-0.20, 0.22]	0.02	0.02
		"	CON+ vs. CXT-	0.28	0.78	91.27	[-0.17, 0.23]	0.06	0.06
		"	CON+ vs. CS-ave.	0.14	0.89	91.95	[-0.18, 0.21]	0.03	0.03
Pupil dilation	Placebo vs. Doxycycline	all	CON+ vs. CON-	-0.52	0.61	86.82	[-0.12, 0.07]	-0.10	-0.10
		"	CON+ vs. CON-ER	-0.60	0.55	86.27	[-0.13, 0.07]	-0.12	-0.12
		"	CON+ vs. CXT-EA	-1.31	0.19	81.32	[-0.18, 0.04]	-0.27	-0.26
		"	CON+ vs. CXT-	-0.03	0.97	86.39	[-0.11, 0.11]	-0.01	-0.01
		"	CON+ vs. CS-ave.	-0.67	0.50	83.15	[-0.13, 0.06]	-0.14	-0.14

Table S4
SEBR recall paired t-test CS+/CS- (CS- 1,2,3,4 and CS- average), not corrected for multiple comparisons

Group	subsets	Conditions	t-statistic	p	df	95% CI	cohen's d	hedge's g
Placebo	1	CON+ vs. CON-	2.93	0.005*	47	[0.09, 0.48]	0.41	0.41
	1	CON+ vs. CON-ER	2.86	0.006*	47	[0.06, 0.37]	0.40	0.40
	1	CON+ vs. CXT-EA	1.75	0.086	47	[-0.03, 0.41]	0.25	0.24
	1	CON+ vs. CXT-	1.75	0.087	47	[-0.03, 0.49]	0.25	0.24
	1	CON+ vs. CS-ave.	2.62	0.012*	47	[0.05, 0.41]	0.37	0.36
	1-2	CON+ vs. CON-	3.24	0.002*	49	[0.08, 0.36]	0.46	0.45
	1-2	CON+ vs. CON-ER	3.41	0.001*	49	[0.09, 0.37]	0.48	0.47
	1-2	CON+ vs. CXT-EA	2.15	0.036*	49	[0.01, 0.35]	0.30	0.30
	1-2	CON+ vs. CXT-	3.02	0.004*	49	[0.09, 0.44]	0.43	0.42
	1-2	CON+ vs. CS-ave.	3.39	0.001*	49	[0.09, 0.36]	0.48	0.47
Doxycycline	1	CON+ vs. CON-	3.62	0.001*	45	[0.26, 0.90]	0.52	0.51
	1	CON+ vs. CON-ER	2.52	0.015*	46	[0.08, 0.70]	0.36	0.36
	1	CON+ vs. CXT-EA	4.10	<.001*	45	[0.34, 0.99]	0.59	0.58
	1	CON+ vs. CXT-	3.93	<.001*	45	[0.31, 0.97]	0.57	0.56
	1	CON+ vs. CS-ave.	3.84	<.001*	46	[0.27, 0.86]	0.55	0.55
	1-2	CON+ vs. CON-	3.63	0.001*	47	[0.24, 0.83]	0.52	0.51
	1-2	CON+ vs. CON-ER	2.54	0.014*	47	[0.07, 0.57]	0.37	0.36
	1-2	CON+ vs. CXT-EA	3.04	0.004*	46	[0.15, 0.76]	0.44	0.43
	1-2	CON+ vs. CXT-	3.72	0.001*	46	[0.23, 0.78]	0.54	0.53
	1-2	CON+ vs. CS-ave.	3.58	0.001*	47	[0.21, 0.74]	0.52	0.51

Table S5
SEBR recall independent t-test between CS+/CS- (CS- 1,2,3,4 and CS- average),
difference for placebo and doxycycline group, not corrected for multiple comparisons

Measure	Group	subsets	Conditions	t-statistic	p	df	95% CI	cohen's d	hedge's g
SEBR	Placebo vs. Doxycycline	1	CON+ vs. CON-	1.58	0.12	92	[-0.08, 0.66]	0.32	0.32
		1	CON+ vs. CON-ER	1.00	0.32	93	[-0.17, 0.51]	0.20	0.20
		1	CON+ vs. CXT-EA	2.42	0.017*	92	[0.08, 0.85]	0.49	0.49
		1	CON+ vs. CXT-	2.00	0.049*	92	[0.00, 0.82]	0.40	0.40
		1	CON+ vs. CS-ave.	1.95	0.054	93	[-0.01, 0.67]	0.39	0.39
		1-2	CON+ vs. CON-	1.95	0.054	96	[-0.01, 0.63]	0.39	0.39
		1-2	CON+ vs. CON-ER	0.62	0.54	96	[-0.19, 0.37]	0.13	0.12
		1-2	CON+ vs. CXT-EA	1.62	0.11	95	[-0.06, 0.61]	0.33	0.32
		1-2	CON+ vs. CXT-	1.52	0.13	95	[-0.07, 0.56]	0.31	0.30
		1-2	CON+ vs. CS-ave.	1.69	0.093	96	[-0.04, 0.54]	0.34	0.34

Table S6
Pupil dilation recall paired t-test CS+/CS- (CS- 1,2,3,4 and CS- average), not corrected for multiple comparisons

Group	subsets	Conditions	t-statistic	p	df	95% CI	cohen's d	hedge's g
Placebo	1	CON+ vs. CON-	2.88	0.006*	48	[0.07, 0.37]	0.40	0.40
	1	CON+ vs. CON-ER	3.99	<.001*	45	[0.13, 0.41]	0.56	0.55
	1	CON+ vs. CXT-EA	4.95	<.001*	47	[0.25, 0.59]	0.69	0.68
	1	CON+ vs. CXT-	4.46	<.001*	46	[0.17, 0.46]	0.62	0.61
	1	CON+ vs. CS-ave.	5.41	<.001*	48	[0.20, 0.44]	0.76	0.75
	1-2	CON+ vs. CON-	4.07	<.001*	49	[0.13, 0.37]	0.57	0.56
	1-2	CON+ vs. CON-ER	4.92	<.001*	49	[0.18, 0.43]	0.69	0.68
	1-2	CON+ vs. CXT-EA	6.41	<.001*	49	[0.30, 0.58]	0.90	0.88
	1-2	CON+ vs. CXT-	5.50	<.001*	49	[0.22, 0.48]	0.77	0.76
	1-2	CON+ vs. CS-ave.	6.34	<.001*	50	[0.24, 0.46]	0.89	0.87
Doxycycline	1	CON+ vs. CON-	4.02	<.001*	45	[0.14, 0.41]	0.59	0.58
	1	CON+ vs. CON-ER	6.45	<.001*	45	[0.23, 0.44]	0.94	0.93
	1	CON+ vs. CXT-EA	4.87	<.001*	45	[0.25, 0.59]	0.71	0.70
	1	CON+ vs. CXT-	4.44	<.001*	46	[0.18, 0.47]	0.65	0.64
	1	CON+ vs. CS-ave.	5.83	<.001*	46	[0.22, 0.45]	0.85	0.84
	1-2	CON+ vs. CON-	4.39	<.001*	46	[0.14, 0.38]	0.64	0.63
	1-2	CON+ vs. CON-ER	6.57	<.001*	46	[0.22, 0.41]	0.96	0.94
	1-2	CON+ vs. CXT-EA	6.60	<.001*	46	[0.31, 0.58]	0.96	0.95
	1-2	CON+ vs. CXT-	5.52	<.001*	46	[0.22, 0.47]	0.81	0.79
	1-2	CON+ vs. CS-ave.	6.58	<.001*	46	[0.24, 0.45]	0.96	0.94

Table S7
Pupil dilation recall independent t-test between CS+/CS- (CS- 1,2,3,4 and CS- average),
difference for placebo and doxycycline group, not corrected for multiple comparisons

Measure	Group	subsets	Conditions	t-statistic	p	df	95% CI	cohen's d	hedge's g
Pupil dilation	Placebo vs. Doxycycline	1	CON+ vs. CON-	0.59	0.56	93	[-0.14, 0.26]	0.12	0.12
		1	CON+ vs. CON-ER	0.77	0.44	90	[-0.10, 0.24]	0.16	0.15
		1	CON+ vs. CXT-EA	0.03	0.98	92	[-0.24, 0.24]	0.01	0.01
		1	CON+ vs. CXT-	0.08	0.94	92	[-0.19, 0.21]	0.02	0.02
		1	CON+ vs. CS-ave.	0.20	0.84	94	[-0.15, 0.18]	0.04	0.04
		1-2	CON+ vs. CON-	0.15	0.88	95	[-0.16, 0.18]	0.03	0.03
		1-2	CON+ vs. CON-ER	0.14	0.89	95	[-0.14, 0.17]	0.03	0.03
		1-2	CON+ vs. CXT-EA	0.07	0.95	95	[-0.19, 0.20]	0.01	0.01
		1-2	CON+ vs. CXT-	-0.08	0.94	95	[-0.19, 0.17]	-0.02	-0.02
		1-2	CON+ vs. CS-ave.	-0.06	0.95	96	[-0.16, 0.15]	-0.01	-0.01

Table S8
Re-learning paired t-test CS+/CS- (CS- 1,2,3,4 and CS- average), not corrected for multiple comparisons

Measure	Group	subsets	Conditions	t-statistic	p	df	95% CI	cohen's d	hedge's g
SCR DCM	Placebo	all	CON+ vs. CON-	5.00	<.001*	49.99	[0.56, 1.30]	0.72	0.71
		"	CON+ vs. CON-ER	5.17	<.001*	48.8	[0.58, 1.33]	0.75	0.73
		"	CON+ vs. CXT-EA	4.90	<.001*	49.32	[0.54, 1.28]	0.71	0.70
		"	CON+ vs. CXT-	4.67	<.001*	49.94	[0.49, 1.24]	0.67	0.66
		"	CON+ vs. CS-ave.	5.00	<.001*	47	[0.55, 1.28]	0.72	0.71
	Doxycycline	all	CON+ vs. CON-	3.91	<.001*	47	[0.25, 0.79]	0.59	0.58
		"	CON+ vs. CON-ER	3.53	0.001*	48	[0.21, 0.75]	0.53	0.52
		"	CON+ vs. CXT-EA	3.96	<.001*	48	[0.26, 0.81]	0.60	0.59
		"	CON+ vs. CXT-	4.23	<.001*	46	[0.30, 0.83]	0.64	0.63
		"	CON+ vs. CS-ave.	4.00	<.001*	43	[0.26, 0.79]	0.60	0.59
Pupil dilation	Placebo	all	CON+ vs. CON-	5.95	<.001*	62	[0.23, 0.46]	0.90	0.88
		"	CON+ vs. CON-ER	6.05	<.001*	66	[0.24, 0.48]	0.91	0.90
		"	CON+ vs. CXT-EA	2.61	0.011*	68	[0.04, 0.28]	0.39	0.39
		"	CON+ vs. CXT-	2.30	0.024*	69	[0.02, 0.26]	0.35	0.34
		"	CON+ vs. CS-ave.	4.47	<.001*	55	[0.14, 0.36]	0.67	0.66
	Doxycycline	all	CON+ vs. CON-	5.79	<.001*	57.95	[0.21, 0.44]	0.88	0.87
		"	CON+ vs. CON-ER	5.99	<.001*	62.73	[0.23, 0.46]	0.91	0.90
		"	CON+ vs. CXT-EA	2.90	0.005*	66.56	[0.05, 0.29]	0.44	0.43
		"	CON+ vs. CXT-	2.73	0.008*	61.95	[0.04, 0.27]	0.42	0.41
		"	CON+ vs. CS-ave.	4.53	<.001*	54.71	[0.14, 0.36]	0.69	0.68

Table S9
Re-learning independent t-test between CS+/CS- (CS- 1,2,3,4 and CS- average),
difference for placebo and doxycycline group, not corrected for multiple comparisons

Measure	Group	subsets	Conditions	t-statistic	p	df	95% CI	cohen's d	hedge's g
SCR	Placebo vs. Doxycycline	all	CON+ vs. CON-	-1.72	0.090	84.39	[-0.88, 0.06]	-0.35	-0.35
		"	CON+ vs. CON-ER	-2.13	0.036*	82.65	[-0.92, -0.03]	-0.43	-0.43
		"	CON+ vs. CXT-EA	-1.58	0.12	82.48	[-0.84, 0.10]	-0.32	-0.32
		"	CON+ vs. CXT-	-1.38	0.17	86.48	[-0.74, 0.13]	-0.28	-0.28
		"	CON+ vs. CS-ave.	-1.73	0.087	83.66	[-0.84, 0.06]	-0.35	-0.35
Pupil dilation	Placebo vs. Doxycycline	all	CON+ vs. CON-	-0.24	0.81	85	[-0.16, 0.12]	-0.05	-0.05
		"	CON+ vs. CON-ER	-0.11	0.91	85	[-0.15, 0.13]	-0.02	-0.02
		"	CON+ vs. CXT-EA	0.23	0.82	85	[-0.14, 0.18]	0.05	0.05
		"	CON+ vs. CXT-	0.18	0.86	85	[-0.14, 0.17]	0.04	0.04
		"	CON+ vs. CS-ave.	0.03	0.98	84.99	[-0.14, 0.14]	0.01	0.01

Tabel S10

LME Analysis of pupil dilation and skin conductance for fear acquisition and re-learning including CS+/US+ trials. Significant effects are marked with '*'.

**Fear acquisition**	Pupil dilation			Skin Conductance		
**Effect**	*F-value*	*df*	*p-value*	*F-value*	*df*	*p-value*
Drug (doxycycline/placebo)	0.18	1, 8246	0.67	0.05	1, 8681	0.82
Trial number	123.17	1, 8246	<.001*	572.05	1, 8681	<.001*
Condition (CS+/CS-$_{1,2,3,4}$)	193.87	4, 8246	<.001*	11.54	4, 8681	<.001*
Drug x Trial	3.36	1, 8246	0.067	3.01	1, 8681	0.083
Drug x Condition	5.06	4, 8246	<.001*	0.11	4, 8681	0.98
Trial x Condition	5.04	4, 8246	<.001*	1.81	4, 8681	0.12
Drug x Trial x Condition	4.80	4, 8246	<.001*	0.98	4, 8681	0.41

**Fear re-learning**	Pupil dilation			Skin Conductance		
**Effect**	*F-value*	*df*	*p-value*	*F-value*	*df*	*p-value*
Drug (doxycycline/placebo)	0.91	1, 3894	0.34	4.11	1, 4152	0.043*
Trial number	8.90	1, 3894	0.003*	258.68	1, 4152	<.001*
Condition (CS+/CS-$_{1,2,3,4}$)	76.58	4, 3894	<.001*	0.33	4, 4152	0.86
Drug x Trial	4.10	1, 3894	0.043*	8.54	1, 4152	0.003*
Drug x Condition	0.31	4, 3894	0.87	3.99	4, 4152	0.003*
Trial x Condition	1.20	4, 3894	0.31	1.40	4, 4152	0.23
Drug x Trial x Condition	0.35	4, 3894	0.85	0.67	4, 4152	0.62
